# Periconceptional multiple-micronutrient supplementation and placental function in rural Gambian women: a double-blind, randomized, placebo-controlled trial^1^

**DOI:** 10.3945/ajcn.113.072413

**Published:** 2015-11-11

**Authors:** Stephen Owens, Ruchi Gulati, Anthony J Fulford, Fatou Sosseh, Fiona C Denison, Bernard J Brabin, Andrew M Prentice

**Affiliations:** 2 Children's Unit, Northumbria Healthcare NHS Foundation Trust, North Shields, United Kingdom;; 3 Medical Research Council (MRC) International Nutrition Group, London School of Hygiene and Tropical Medicine, London, United Kingdom, and MRC Keneba, Fajara, The Gambia;; 4 MRC Centre for Reproductive Health, The Queen’s Medical Research Institute, Edinburgh, United Kingdom; and; 5 Child and Reproductive Health Group, Liverpool School of Tropical Medicine, Liverpool, United Kingdom

**Keywords:** periconceptional, micronutrients, pregnancy, placenta, Africa, birth weight, fetal growth

## Abstract

**Background:** Maternal micronutrient deficiencies are commonly associated with clinical indicators of placental dysfunction.

**Objective:** We tested the hypothesis that periconceptional multiple-micronutrient supplementation (MMS) affects placental function.

**Design:** We conducted a double-blind, randomized, placebo-controlled trial of MMS in 17- to 45-y-old Gambian women who were menstruating regularly and within the previous 3 mo. Eligible subjects were pre–randomly assigned to supplementation with the UNICEF/WHO/United Nations University multiple micronutrient preparation (UNIMMAP) or placebo on recruitment and until they reached their first antenatal check-up or for 1 y if they failed to conceive. Primary outcome measures were midgestational indexes of utero-placental vascular-endothelial function [ratio of plasminogen-activator inhibitor (PAI) 1 to PAI-2 and mean uterine-artery resistance index (UtARI)] and placental active transport capacity at delivery [fetal to maternal measles antibody (MMA) ratio].

**Results:** We recruited 1156 women who yielded 415 pregnancies, of which 376 met all of the inclusion criteria. With adjustment for gestational age at sampling, there were no differences in PAI-1 to PAI-2 or MMA ratios between trial arms, but there was a 0.02-unit reduction in UtARI between 18 and 32 wk of gestation (95% CI: −0.03, −0.00; *P* = 0.040) in women taking UNIMMAP.

**Conclusions:** Placental vascular function was modifiable by periconceptional micronutrient supplementation. However, the effect was small and supplementation did not further affect other variables of placental function. This trial was registered at www.controlled-trials.com as ISRCTN 13687662.

## INTRODUCTION

Micronutrient malnutrition is a common, global public health problem, particularly for women living in poverty (1). Poor-quality diets, low in animal-sourced foods, can lead to composite micronutrient deficiencies (2) and adverse pregnancy outcomes (3). Previous attempts to attenuate these effects through multiple-micronutrient supplementation (MMS)^6^ have resulted in modest increases in birth weight but no consistent effect on maternal or perinatal morbidity (4). However, previous studies may have started MMS too late to effect substantial changes in pregnancy outcomes. The periconceptional period (before conception and up to 12 wk of gestation) is arguably the optimal exposure window for nutritional interventions because it encompasses several critical gestational processes including implantation and placentation (6). A recent systematic review identified only 9 micronutrient trials that specifically targeted the periconceptional period and most were designed to investigate the impact of supplementation on the incidence of birth defects (5). The effect of early pregnancy micronutrient supplementation on the developing placenta has been largely ignored.

The placenta is a complex endocrine organ integral to the regulation of materno-fetal metabolism, physiology, and growth (7). Placental vascularization during the first half of pregnancy defines later placental transfer capacity and ultimately the fetal growth trajectory and pregnancy outcome (8). This early period of placental angiogenesis is characterized by strictly regulated oxidative stress and inflammation, endothelial remodeling, and rapid cell turnover (9). Inadequate maternal and/or fetal micronutrient supply could compromise any of these processes, impairing angiogenesis and leading to placental dysfunction, fetal growth restriction (FGR), and preterm birth (PTB) (10).

Several micronutrients have been implicated in shaping placental function, especially folic acid, vitamin D, antioxidant vitamins, calcium, and iron (11). The mechanisms of action are largely speculative but include molecular roles for several micronutrients in the control of free radical production in hypoxic early-placenta vasculature (10). Yet, it is unlikely that a single-nutrient intervention will improve placental function and fetal growth in most pregnancies, especially where combined deficiencies are common. On the contrary, the need for a more balanced, composite approach is evidenced by the finding that universal folic acid supplementation of pregnant women in areas where vitamin B-12 deficiency is common may program diabetes risk in children (12). We conducted a randomized, double-blinded, placebo-controlled trial of periconceptional MMS in rural Gambian women to test the hypothesis that periconceptional micronutrient status modulates placental function in later pregnancy.

## METHODS

### Study design

The study was a double-blind, randomized, placebo-controlled trial of periconceptional MMS assessed on 3 primary outcomes. The first was the ratio of maternal plasminogen activation inhibitor (PAI) 1 (a marker of endothelial activation) to PAI-2 (a marker of placental function) at 18–22 wk of gestation (13). In a healthy pregnancy, the concentrations of both biomarkers should increase progressively, but their ratio should decline as placental mass, and PAI-2 production, increases (14).

The second primary outcome was uterine-artery Doppler waveform at 18–22 wk of gestation [a surrogate marker of placental perfusion that correlates with trophoblast invasion (15)]. The uterine-artery pulsatility index (UtAPI) and uterine-artery resistance index (UtARI) were also measured at 28–32 wk of gestation, and diastolic notching was noted. These indexes quantify systolic and diastolic components of the flow velocity waveform in a specific blood vessel over a single cardiac cycle. The higher the values, the greater the downstream vascular resistance. The third primary outcome was the ratio of the delivery concentrations of fetal to maternal measles antibody (MMA), which was used as a proxy marker of placental transport capacity.

### Intervention

The micronutrient supplement (Lomapharm) was a coated tablet containing 15 vitamins and trace elements, formulated to a composition specified by the UNICEF/WHO/United Nations University (UNICEF/WHO/UNU) for use by pregnant women in the developing world and known as the UNICEF/WHO/UNU international multiple micronutrient preparation (UNIMMAP) (16). UNIMMAP contains the following: vitamin A (800 retinol equivalents), vitamin D (200 IU), vitamin E (10 mg), vitamin C (70 mg), thiamin (1.4 mg), riboflavin (1.4 mg), niacin (18 mg), pyridoxine (1.9 mg), cobalamin (2.6 mg), folic acid (400 μg), iron (30 mg), zinc (15 mg), copper (2 mg), selenium (65 μg), and iodine (150 μg). The placebo was manufactured to be indistinguishable from the supplement (Lomapharm).

### Study setting

The study took place between March 2006 and June 2008 in the Kiang West region of The Gambia among 33 villages under demographic surveillance by the United Kingdom Medical Research Council (MRC) field station staff at Keneba. Kiang West comprises 750 km^2^ of savannah scrub and is bordered on 3 sides by the River Gambia and its tributaries and on the fourth by a partially surfaced road. The population consists of ∼14,000 individuals, predominantly ethnic Mandinka, who live by subsistence farming. Nutritional status and morbidity patterns in this community have been well described and are largely defined by a distinct tropical seasonality, with a long dry season from November to June followed by a period of intense and daily rainfall between July and October (17–19). Local HIV seroprevalence at the time of the study was ∼1% (20). In this area, the incidence of low birth weight was 13%, PTB was 12%, and 25% of newborns were small for gestational age (<10th centile weight for gestational age) with FGR (18).

Local maternal and child primary health care is provided by government nurse trekking teams, supported by the clinical staff of MRC Keneba. Most women deliver at home under the supervision of traditional birth attendants (TBAs) who undergo basic training in clean birth practices and recognition of common pregnancy complications (21). Secondary and tertiary health care services are provided at the Royal Victoria Teaching Hospital (RVTH) in Banjul, which is 4 h by road from Keneba.

### Selection of subjects

Women aged 17–45 y and residing in Kiang West during the recruitment phase of the study and who were registered in the MRC Keneba demographic database and not concurrently enrolled in other intervention studies were eligible to take part in the trial. Eligible women (and their guardians for those <18 y old) were invited to attend a recruitment clinic in their home villages. They were provided with oral and printed study information by a nurse-midwife (NM) and asked to give written consent if they wished to take part. Those who used no contraception, were not known to be pregnant, and had experienced menses in the past 3 mo and were not breastfeeding were included. Those who were severely anemic (hemoglobin concentration <7 g/dL) were treated before being considered for re-recruitment at a later date. Women with a subsequent multiple pregnancy or obvious fetal abnormality were excluded, although they were clinically followed because nonnutritional factors determine placental function in such pregnancies (22).

### Randomization

Eligible subjects identified in the demographic survey were randomly assigned before recruitment to 1 of 4 color-coded groups. An independent researcher allocated 2 color codes to intervention and 2 to placebo groups and held the allocation key. Randomization was completed by an MRC Keneba statistician (AJF) by using computer-generated permuted blocks of 8, stratified by maternal age. Researchers involved in recruitment, data collection, or analysis were blind to subjects’ color codes, and all staff and participants were blind to the allocation key.

### Recruitment

The NM recorded a brief obstetric, medical, and educational history and collected a 10-mL venous blood sample from each participant (Sarstedt Monovette). Whole blood was analyzed for hematology and the presence of malaria parasites. Plasma was separated within 4 h of venipuncture, frozen at −80°C, and later analyzed for vitamin A and ferritin concentrations. Weight, height, left midupper arm circumference (MUAC), and systolic/diastolic blood pressure were measured by a trained fieldworker. Women were issued tablets corresponding to their randomized color-coded intervention group and were asked to take 1 tablet daily until told to stop by a member of the trial team.

### Supplementation

Participants were seen at least fortnightly by a fieldworker in their village and were resupplied with tablets according to their color groups. The number of tablets apparently consumed between visits was used as a measure of compliance (compliance = number of tablets apparently consumed/number of days enrolled in study). Information on morbidity and menstrual patterns was recorded. Women were asked to notify fieldworkers of missed menstrual periods and to provide urine samples for pregnancy testing by an NM when pregnancy was suspected (QuickVue One-Step hCG Urine Test; bioMérieux). With a positive pregnancy test result, the woman’s study intervention was withdrawn and replaced with 60 mg elemental iron and 250 μg folic acid daily, in accordance with Gambian national antenatal policy. These women were referred to the study antenatal clinic (ANC) at MRC Keneba for pregnancy confirmation and follow-up. Those without a positive pregnancy test result continued with supplementation until July 2007 (a period of 12–14 mo), at which time the supplementation was discontinued. Hematologic outcomes for nonpregnant participants are reported elsewhere (23). Women who migrated from the study area for 6 consecutive weeks were defined as lost to follow-up. Fetal deaths were classified as miscarriages (spontaneous loss of pregnancy before 24 completed weeks of gestation) or stillbirths (no signs of life at birth after 24 completed weeks of gestation). These were reported by TBAs and cross-checked by the fieldworker and/or the NM. In cases of uncertainty, confirmatory sonography and urinary pregnancy testing were completed at the ANC in MRC Keneba.

### Pregnancy follow-up

Viable pregnancy was confirmed and dated by using transabdominal ultrasound at the ANC booking clinic. Women without a viable pregnancy at the ANC were assumed to have miscarried and were censored from the study. Women with multiple pregnancies or fetal anomalies were also censored. All women were offered continuing clinical care, and those who required specific obstetric management were referred to RVTH.

Women with sonographically dated singleton pregnancies were invited and offered transport to attend the ANC for complete clinical review at 18–22 wk and 28–32 wk, including anthropometric measurements, urinalysis, blood pressure assessment, external obstetric examination, detailed fetal sonography, and venipuncture. These tasks were completed by the NM, and sonography was completed by one of the authors (SO). Pregnancies of >24 wk of gestation at booking could not be accurately dated by ultrasound and were dated by maternally recalled last menstrual period (LMP). Because maternally recalled LMP is imprecise in this population (24), a restricted analysis of delivery outcomes alone was used for this subgroup.

At ANC booking, a 5-mL blood sample was drawn and analyzed for hemoglobin concentration, presence of malaria parasites, and syphilis serology. As required by the Gambian Government Ethics Committee, each subject was offered serologic testing for HIV, with pre- and posttest counseling. HIV-positive participants were referred to the MRC HIV clinical service in Fajara. Women who delivered before attendance at the HIV clinic were offered maternal and infant nevirapine prophylaxis, in keeping with the national guideline at the time.

An uncuffed 10-mL venous blood sample was drawn at the 18–22-wk and 28–32-wk review. Aliquots were prepared, stored, and analyzed within 1 h of venipuncture, as described for recruitment samples, except for ferritin concentration, which was not measured at 18–22 wk. Additional aliquots of chilled citrate-plasma aliquot and serum were analyzed for PAI-1 and PAI-2 concentrations and placental hormones [human prolactin (hPL) and human chorionic gonadotropin (hCG)], respectively. The subject was given directly observed intermittent preventative treatment with sulfadoxine-pyrimethamine at 18–22 wk and 28–32 wk.

TBAs reported impending deliveries to their local resident fieldworker. A 10-mL cord blood sample taken from a large vein on the fetal side of the placenta of live-born infants. The placenta was transported as soon as it was collected to MRC Keneba, cleaned, the membranes trimmed, and the cord cut close to its insertion. Placental weight was recorded to the nearest 10 g by using a digital balance. Cord serum and whole-blood aliquots were prepared. The concentration of cord serum MMA was measured. Whole blood was analyzed for hematologic variables and the presence of malaria parasites.

Within 72 h of delivery, an NM examined the mother and infant. Maternal weight, MUAC, urinalysis, and blood pressure were recorded. An uncuffed 10-mL venous blood sample was drawn from the mother and aliquots prepared and analyzed as for the cord samples. Neonatal anthropometric measurements were recorded. Dubowitz scoring was conducted to improve the detection of preterm infants, as part of standard clinical risk assessment.

### Measurements

Maternal height, weight, MUAC, and blood pressure were measured by pairs of fieldworkers at recruitment and by 1 of 2 NMs at scheduled antenatal visits and at delivery. Standard techniques were used for each measurement. Weight was measured (to the nearest 0.1 kg) by using daily calibrated, digital scales (Tanita Corporation). Height (to the nearest 0.1 cm) was measured by using a daily calibrated stadiometer (Leicester height measure; Seca) and BMI was estimated [weight (kg)/height (m)^2^]. Underweight was defined as a BMI (in kg/m^2^) <18.5. MUAC (to the nearest mm; left arm) was measured with graduated tapes (Henley Medical Supplies). Triplicate blood pressure was measured (automated Omron 705IT device; Omron) and measurements replicated to within 5 mm Hg. Hypertension was defined as systolic blood pressure >139 mm Hg or diastolic blood pressure >89 mm Hg.

Neonatal anthropometric measurements were performed by 1 of 2 NMs. Birth weights were measured (to the nearest 20 g) with sling and portable spring balances (CMS Weighing Equipment), and these were regularly checked with standard weights. Birth length (to the nearest 5 mm) was measured by using neonatal length mats (TALC Teaching Aids). Head circumference (to the nearest mm) was measured with graduated tapes (Henley Medical Supplies). Fieldworkers and NMs were trained and cross-compared in anthropometric techniques as part of their employment induction with the MRC, which was repeated at the start of this study. Refresher training was undertaken regularly.

Hemoglobin measurements at pregnancy booking used a hemoglobinometer (HemoCue B). Analyses on venipuncture samples included hemoglobin concentration, white blood cell count, mean cell volume, mean cell hemoglobin, and reticulocyte count (Cel-Dyn 3700 Analyzer; Abbott Diagnostics). Anemia was defined as hemoglobin <12 g/dL in nonpregnant women and <11 g/dL in pregnant women. Malaria blood films were prepared and stained with Giemsa stain, and 100 high-power microscopic fields were examined to determine parasite count against 200 white blood cells. PAI-1 and PAI-2 antigen concentrations were measured with mouse-monoclonal antibody–based ELISAs (Elitest; Hyphen-BioMed) as was MMA in maternal and fetal blood (IBL International), hPL, and hCG (Addenbrookes Hospital).

Vitamins A and E were measured by HPLC (25). Vitamin A deficiency was defined as a serum retinol concentration <0.7 μmol/L and vitamin E deficiency as a serum α-tocopherol concentration <12 μmol/L. Ferritin was measured by instrumental immunoassay (Dimension Xp; Siemens). Iron deficiency was defined as a plasma ferritin concentration <15 μg/L.

Obstetric sonography was conducted by one of the authors (SO) who had certified training in standard methods (25). The ultrasound machine was an ACUSON Antares with a CH6-2 (5.71-MHz) transducer (Siemens). Gestational age was derived from sonographic measurement of the fetal crown-rump length or biparietal diameter. At the 18–22-wk and 28–32-wk review, fetal biometry and utero-placental blood flow were assessed by sonography and Doppler velocimetry of the right and left uterine arteries (UtAs), umbilical artery, and fetal middle cerebral artery (MCA) waveforms. The resistance and pulsatility indexes were calculated for each vessel and for the peak systolic velocity (PSV) of the MCA. The presence of diastolic “notching” in either of the UtAs was recorded. A mean UtARI >0.55 and bilateral notching, or a mean UtARI >0.65 and unilateral notching, were defined as “high resistance” waveforms (26). Women with high-resistance waveforms were monitored for pre-eclampsia and/or FGR and, if necessary, referred to RVTH. Intraobserver variation in sonographic variables was not estimated.

### Statistical analysis

We estimated that a sample of 200 women in each arm of the trial would generate 90% power to detect a 20% difference in the mean PAI-1 to PAI-2 ratio at 18–22 wk of gestation between supplementation groups at a 5% level of significance. This was based on variance parameters (population mean ratio: 0.40; SD: 0.23) drawn from a trial of antioxidant vitamin supplementation in British women at risk of pre-eclampsia (27) and allowed for 10% loss to follow-up. This sample size had 90% power to detect a change in mean UtARI equivalent to 0.35 SDs at the 5% significance level [assuming a mean UtARI of 0.30; SD: 0.01 (26)].

To detect a difference in mean MMA transplacental transfer ratio of 0.1, with 90% power at the 5% level of significance, ∼80 mother-infant pairs were required in each arm of the trial, assuming a mean transfer ratio of 1.01 and an SD of 0.18 (28). It was considered that this sample size would be readily available within the main pregnancy cohort.

On the basis of MRC Keneba demographic survey data, we assumed that 16% of women aged between 17 and 45 y in this community would conceive in any given year. This suggested that at least 2400 women would be required over the course of the study to satisfy the largest estimate of required sample size, 400 pregnancies. We identified 3206 eligible subjects in the survey.

Data were double-entered into a computerized database (MS-Access; Microsoft) and verified within 48 h of collection. Scheduled data validation checks were made. A copy of the raw data set was maintained with the MRC data management team.

All available data on singleton pregnancies without fetal anomalies were included and analyzed according to the original randomization. Every effort was made to complete the data set for each subject, but specific outcomes on individual subjects were occasionally unavailable (e.g., on women who had withdrawn their consent, migrated out of the study area, or otherwise failed to attend within the predefined timeframe of a particular endpoint). In these cases, the remaining data were included in all other analyses. Continuous data were analyzed by using linear regression, assuming equal variance. Outcomes were regressed on a single predictor, trial arm. To improve the precision of primary analyses, multiple regression was used to adjust for the effects of gestational age at point of sampling, because gestational age is an a priori predictor of all pregnancy outcomes. Evidence for interaction between treatment effect and gestational age at point of sampling was also sought for primary outcome measures. Continuous data that were not normally distributed were log-transformed before analyses of their geometric means. Those that were not rendered normally distributed by this transformation were analyzed by using nonparametric tests. Binary responses were analyzed by logistic regression. Data collected on the same individuals at ≥2 time points were analyzed by using random-effects models fitted by generalized least squares.

Although the study was not powered for subgroup analyses, we went on to assess treatment effects on the primary outcomes by season of conception (wet-hungry compared with dry-harvest) by fitting a season × treatment interaction term into the models. This stratification was justified as an exploratory analysis on the basis of previous data showing that PTB and small-for-gestational-age birth show strongly divergent patterns of seasonality in Kiang West (18). Significance was set at *P* < 0.05. Statistical analyses were carried out by using Stata 9.1 (StataCorp).

### Medical management

All women and their infants were offered free routine clinical services, including infant vaccinations. Transport was provided by MRC ambulance. Malaria parasitemia was treated with chloroquine or quinine and sulfadoxine-pyrimethamine in accordance with national policy. Pre-eclampsia was defined by a gestational systolic blood pressure >140 mm Hg or a diastolic blood pressure >90 mm Hg in a previously normotensive woman, in conjunction with dipstick proteinuria. Women with pre-eclampsia were referred to RVTH for further management.

### Ethics

This trial was approved by the Scientific Coordinating Committee of MRC Laboratories, The Gambia, and by the MRC/Gambian Government Ethics Committee (L2005.111v2 SCC 1000). An independent trial monitor and data safety and monitoring board assessed trial activity at regular intervals, under the auspices of Good Clinical Practice guidelines (29). MRC Keneba offers free primary health care in collaboration with the Gambian Government Lower River Divisional Health Team. Apart from the structured clinical contacts outlined (during which subjects were given transport and a meal), no additional benefits were provided to trial participants. The trial was registered as ISRCTN 13687662 (www.controlled-trials.com/isrctn/pf/13687662).

## RESULTS

### Characteristics of study population

**FIGURE 1:**
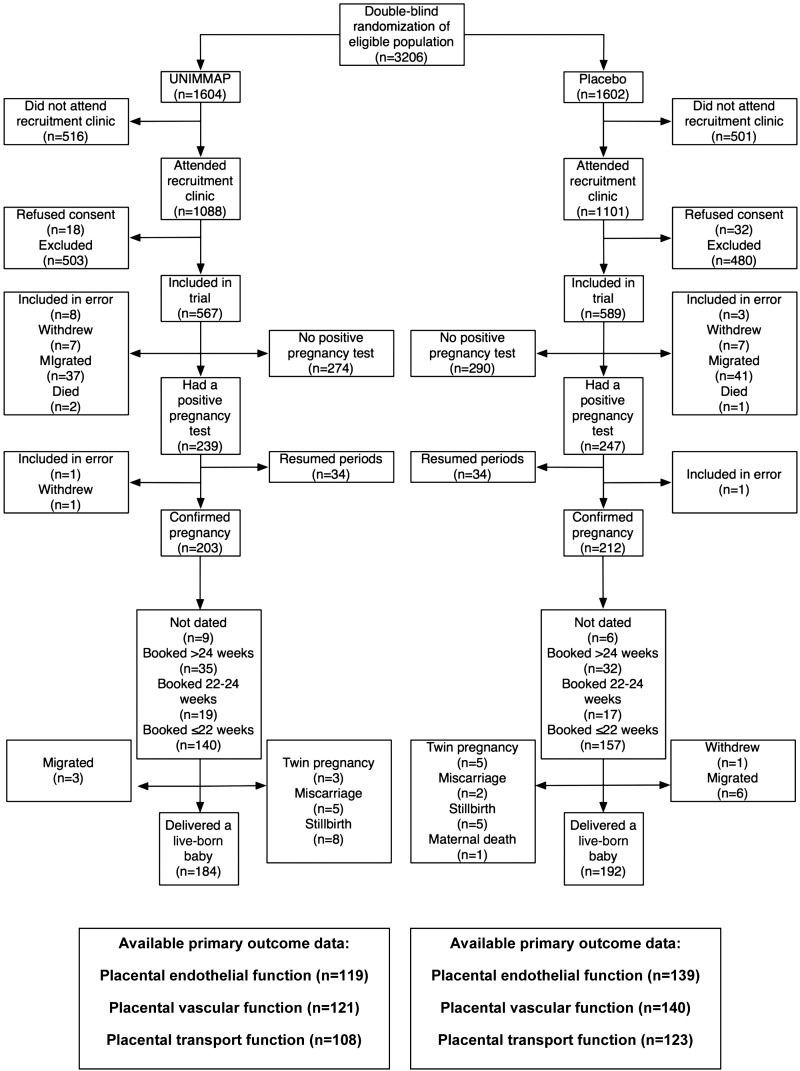
CONSORT (Consolidated Standards of Reporting Trials) diagram. UNIMMAP, UNICEF/WHO/United Nations University international multiple micronutrient preparation.

The study profile [CONSORT (Consolidated Standards of Reporting Trials) diagram] is shown in **Figure 1**. After prescreening the demographic surveillance database, we identified and randomly assigned 3206 women who were eligible for inclusion in the trial on the basis of age, residency, and lack of involvement in other intervention studies. Of these, 2189 (68.2%) attended a recruitment clinic to be screened for exclusion criteria from March to May 2006 (*n* = 1337), May–June 2006 (*n* = 610), and June–July 2006 (*n* = 242). We recruited 1156 women, 567 (49%) in the UNIMMAP arm and 589 (51%) in the placebo arm. Reasons for exclusion included refusal to consent (*n* = 50), known or suspected pregnancy (*n* = 205), breastfeeding an infant (*n* = 362), failure to menstruate in the previous 3 mo (*n* = 393), and use of depot contraception (*n* = 23). Two women were withdrawn because they were later discovered to be in late pregnancy, having booked for standard antenatal care before recruitment, and one pregnant woman withdrew consent.

The median age of participants was 28.8 (IQR: 21.7–36.7) y, and 213 (18.4%) were teenagers. Most were illiterate; 936 (81.0%) had never attended primary school. Parity ranged between 0 and 14, and 294 women were nulliparous (25.4%). A history of pre-eclampsia was elicited from 3.1%, and 22.7% reported a previous pregnancy loss. Overall, 14.8% were underweight and 44.2% iron deficient. The prevalence of anemia (hemoglobin <12 g/dL) was 50.5%, with 0.6% who were severely anemic (hemoglobin <7 g/dL). Almost all of the women (97.2%) reported sleeping under a bed net the previous night. The prevalence of malaria parasitemia was 0.9%. Vitamin A deficiency occurred in 4.0% and vitamin E deficiency in 1.2% of women.

There were 11 women who were excluded after the late identification of exclusion criteria, 14 withdrew without giving a reason, 78 nonpregnant women migrated from the study area, and 3 women died. Those lost to follow-up during supplementation (*n* = 106) were younger than the remaining women (*n* = 1050) [23.0 (IQR: 19.0–29.8) compared with 29.7 (IQR: 21.9–36.8) y; *P* < 0.001], were less likely to have children (50.0% compared with 77.1%; OR: 0.30; 95% CI: 0.20, 0.45; *P* < 0.001), and more likely to have schooling (42.5% compared with 16.7%; OR: 3.69; 95% CI: 2.43, 5.60; *P* < 0.001). There were no differences in baseline nutritional characteristics.

### Supplementation

There was no difference in participant attendance frequency at supplementation clinics between women in the 2 study arms [UNIMMAP: 72% (IQR: 50–85%); placebo: 71% (IQR: 48–85%; *P* = 0.307, Wilcoxon’s rank-sum test]. On the basis of tablet counts, intervention compliance was 88% (IQR: 65–100%) in the UNIMMAP arm compared with 86% (IQR: 63–100%) in the placebo arm (*P* = 0.158, Wilcoxon’s rank-sum test).

### Pregnancy booking

There were 486 women with positive pregnancy tests during the study. The baseline characteristics of these women were similar in both arms of the trial, although more women in the placebo arm had undergone secular schooling (**Table 1**).The median time with supplementation before the generation of a positive pregnancy test was 24.1 wk (IQR: 13.1–43.1), with no difference between study arms (*P* = 0.943). Pregnancies were clinically confirmed in 203 of 567 (35.8%) of those receiving UNIMMAP and 212 of 589 (36.0%) of those receiving placebo. It was not possible to differentiate early miscarriages from false-positive pregnancy test results for 68 women who resumed menstruation before clinical confirmation of pregnancy. However, there was no difference in the odds of unconfirmed pregnancy between arms (OR for UNIMMAP: 1.04; 95% CI: 0.63, 1.74; *P* = 0.868).

**TABLE 1:** Baseline characteristics of participants who later returned positive pregnancy tests^1^

	UNIMMAP (*n* = 239)		Placebo (*n* = 247)	
Variable	*n*	Value	*n*	Value
Maternal age, y	239	28.8 (23.7–36.7)^2^	247	28.9 (23.7–34.8)
Secular schooling, *n* (%)	239	29 (12.1)	247	46 (18.6)^3^
Nulliparity, *n* (%)	239	26 (10.9)	247	25 (10.1)
History of pre-eclampsia, *n* (%)	239	6 (2.5)	247	6 (2.4)
History of pregnancy loss, *n* (%)	239	58 (24.3)	247	66 (26.7)
BMI, kg/m^2^	239	20.7 (19.3–22.9)	247	20.9 (19.4–23.3)
Underweight,^4^ *n* (%)	239	34 (14.2)	247	30 (12.2)
Stunted,^5^ *n* (%)	239	6 (2.5)	247	15 (6.1)
Blood pressure,^6^ mm Hg	239	107 (100–115)	246	108 (101–114)
Retinol, μmol/L	236	1.09 (0.90–1.33)	241	1.16 (0.97–1.36)
Vitamin A deficiency,^7^ *n* (%)	236	11 (4.7)	241	5 (2.1)
α-Tocopherol, μmol/L	236	20.0 (17.6–23.8)	241	20.8 (17.2–24.0)
Vitamin E deficiency,^8^ *n* (%)	236	6 (2.5)	241	2 (0.8)
Ferritin, μg/L	235	17.3 (9.8–28.6)	240	16.9 (9.9–31.3)
Iron deficiency,^9^ *n* (%)	235	78 (33.2)	240	76 (31.7)
Hemoglobin, g/dL	238	11.9 (10.9–12.6)	245	12.0 (11.1–12.8)
Anemia,^10^ *n* (%)	238	124 (52.1)	245	121 (49.4)
CRP, mg/L	235	0.7 (0.3–1.5)	240	0.6 (0.3–1.3)
Malaria, *n* (%)	232	2 (0.9)	240	3 (1.3)

1 CRP, C-reactive protein; UNIMMAP, UNICEF/WHO/United Nations University international multiple micronutrient preparation.

2 Median; IQR in parentheses (all such values).

3 Pearson chi-square = 0.1694, *P* = 0.048.

4 BMI <18.5.

5 Height <151.8 cm.

6 Systolic blood pressure.

7 Retinol <0.7 μmol/L.

8 α-Tocopherol <12 μmol/L.

9 Ferritin <12 μg/L.

10 Hemoglobin <12 g/dL.

Of 415 clinically confirmed pregnancies, 312 were dated sonographically, 88 were dated by LMP, and 15 were undated. On the basis of dating assessments, 105 women were pregnant at recruitment (range: 0–16.4 wk of gestation; median: 5.1 wk). For dated pregnancies, the median time with preconceptional supplementation was 10.9 wk (IQR: −0.3 to +29.4) and with postconceptional supplementation was 11.0 wk (IQR: 7.9–15.8), which did not differ between arms in either period (*P* = 0.922 and 0.428, respectively). Of 295 pregnancies conceived while receiving supplementation, there was no difference in conception rates with UNIMMAP or placebo (HR for UNIMMAP: 0.94; 95% CI: 0.75, 1.18; *P* = 0.613). There were 8 twin pregnancies (3 UNIMMAP, 5 placebo).

### Placental vascular-endothelial function

There were 333 women who booked with a singleton pregnancy on or before 24 wk of gestation by antenatal ultrasound. Of these, 262 agreed to attend for measurement of the primary endpoint at 18–22 wk. There was no difference in PAI-1 or PAI-2 concentrations, or their ratios, between women in either arm (**Table 2**). Similarly, the geometric mean PAI-1 to PAI-2 ratio at 28–32 wk was 0.053 in the UNIMMAP arm (*n* = 125) and 0.052 in the placebo arm (*n* = 130) (difference: +1.6%; 95% CI: −13.6%, +16.9%; *P* = 0.832). Adjustment for gestational age at the point of sampling did not change the effect of supplementation on PAI ratio over the course of pregnancy and there was no evidence of any interaction between treatment effect and gestational age at the point of sampling (*P* = 0.554).

**TABLE 2:** Maternal PAI concentrations and ratio at 18–22 wk of gestation by allocation^1^

	UNIMMAP (*n* = 119)		Placebo (*n* = 139)			
Variable	*n*	Geometric mean (95% CI)	*n*	Geometric mean (95% CI)	Difference estimate (95% CI), %	*P*
PAI-1, ng/mL	119	21.7 (19.8, 23.7)	139	21.0 (19.5, 22.7)	+3.0 (−8.6, +14.7)	0.606
PAI-2, ng/mL	119	332.2 (200.7, 366.9)	138	330.1 (301.6, 361.7)	+0.6 (−12.8, +13.9)	0.935
PAI-1:PAI-2	119	0.07 (0.06, 0.07)	138	0.06 (0.06, 0.07)	+2.6 (−13.3, +18.6)	0.746

1 Significance was assessed by multiple linear regression, adjusted for gestational age. PAI, plasminogen activation inhibitor; UNIMMAP, UNICEF/WHO/United Nations University international multiple micronutrient preparation.

Women taking UNIMMAP had a reduced UtARI (*P* = 0.0499) and UtAPI (*P* = 0.025) compared with women taking placebo at 18–22 wk of gestation (**Table 3**). The PSV in the fetal MCAs was also slower in the UNIMMAP arm (*P* = 0.035), but there were no differences in the indexes of blood flow in the umbilical arteries.

**TABLE 3:** Doppler velocimetry indexes at 18–22 wk of gestation by allocation^1^

	UNIMMAP (*n* = 121)		Placebo (*n* = 140)			
	*n*	Mean ± SD	*n*	Mean ± SD	Difference estimate (95% CI)	*P*
Uterine arteries						
PI	121	1.12 ± 0.29	140	1.20 ± 0.31	−0.08 (−0.16, −0.01)	0.025
RI	121	0.61 ± 0.08	140	0.63 ± 0.08	−0.02 (−0.04, −0.00)	0.050
Umbilical artery						
PI	120	1.36 ± 0.20	139	1.38 ± 0.20	−0.01 (−0.06, +0.04)	0.631
RI	120	0.74 ± 0.07	139	0.74 ± 0.06	−0.00 (−0.02, +0.01)	0.906
Middle cerebral artery						
PI	87	1.41 ± 0.18	102	1.46 ± 0.22	−0.05 (−0.12, +0.01)	0.097
RI	87	0.74 ± 0.07	102	0.76 ± 0.08	−0.02 (−0.04, +0.00)	0.068
PSV, cm/s	85	28.2 ± 4.43	102	29.8 ± 5.31	−1.54 (−2.96, −0.11)	0.035

1 Significance was assessed by multiple linear regression adjusted for gestational age. PI, pulsatility index; PSV, peak systolic velocity; RI, resistance index; UNIMMAP, UNICEF/WHO/United Nations University international multiple micronutrient preparation.

There were 297 women who underwent at least one Doppler study between 18 and 32 wk of gestation. After adjustment for gestational age at the time of measurement, UNIMMAP supplementation reduced the UtARI by a mean of 0.02 units compared with placebo over the course of the pregnancy (18–32 wk) (95% CI: −0.03, −0.00; *P* = 0.040; *n* = 297; 526 observations). There was no evidence of any interaction between treatment effect and gestational age at the point of sampling (*P* = 0.831). In the UNIMMAP arm of the trial, 3 of 129 (2.3%) women had high-resistance Doppler waveforms in the uterine arteries at 28–32 wk of gestation compared with 7 of 136 (5.2%) women in the placebo group (OR: 0.44; 95% CI: 0.11, 1.73; *P* = 0.228).

### Placental transfer capacity

UNIMMAP supplementation had no effect on MMA transfer ratio at delivery compared with placebo (95% CI: −8.4, +22.0; *P* = 0.379; *n* = 231). Adjustment for gestational age at the point of sampling did not affect this finding, and there was no evidence of any interaction between treatment effect and gestational age at the point of sampling (*P* = 0.730). There were no differences in fetal anthropometric indexes between the groups at either of the antenatal time points (data not shown).

### Stratification of primary outcomes by season of conception

There were no significant interactions between effects of seasonality of conception and supplementation on the primary outcomes (PAI ratio: *P* = 0.654; UtARI: *P* = 0.340; placental transfer ratio: *P* = 0.597). Pregnancies conceived in the hungry season were associated with a 27.4% decline in transplacental transfer ratio of MMA (95% CI: 10.8, 44.0; *P* = 0.001), independently of gestational age at delivery and supplementation arm. Seasonality had no independent effects on the other primary outcomes (data not shown).

### Placental endocrine function

There were no differences in geometric mean hCG and hPL concentrations at 18–22 wk of gestation between the arms of the study—hCG: 14.9 (95% CI: 13.1, 17.0) μg/mL in the UNIMMAP group (*n* = 116) compared with 14.9 (95% CI: 13.3, 16.8) μg/mL in the placebo group (*n* = 137) (*P* = 1.000); hPL: 2.52 (95% CI: 2.40, 2.66) μg/mL in the UNIMMAP group compared with 2.66 (95% CI: 2.51, 2.82) μg/mL in the placebo group (*P* = 0.179).

### Birth outcomes

There were 10 confirmed pregnancies with unknown outcomes because of migration or withdrawal before delivery (3 UNIMMAP, 7 placebo). Of 8 twin pregnancies, one in the placebo arm ended in a discordant fetal death. Of singleton pregnancies with known outcomes, 13 of 197 (6.6%) in the UNIMMAP arm and 8 of 200 (4.0%) in the placebo arm ended in fetal death (OR: 1.70; 95% CI: 0.69, 4.19; *P* = 0.252). One fetal death in the placebo group was associated with a maternal death.

Fewer male infants were born to women taking UNIMMAP than to those taking placebo (48.4% compared with 57.8%; *P* = 0.07). The study was not powered to assess the effect of supplementation on neonatal anthropometric measurements, and there were no differences in birth weight, length, head circumference, or placental weight between the groups, before or after adjustment for gestational age at delivery (data not shown). Gestational age distribution at delivery was skewed. Median gestation was 40.3 wk (IQR: 38.7–41.1 wk) in the placebo group and 40.0 wk (IQR: 38.9–41.1 wk) in the UNIMMAP group (*P* = 0.669). However, 10 of 177 (5.6%) infants were born preterm in the UNIMMAP arm compared with 23 of 190 (12.1%) in the placebo arm (OR: 0.43; 95% CI: 0.20, 0.94; *P* = 0.035). Among ultrasound-dated pregnancies, 8 of 150 (5.3%) live-born infants in the placebo arm were preterm compared with 1 of 139 (0.7%) in the UNIMMAP group (OR: 0.13; 95% CI: 0.02, 1.04; *P* = 0.055).

### Maternal and neonatal adverse events

There were 423 of 567 women (74.6%) in the UNIMMAP arm of the study who reported at least one adverse event compared with 442 of 589 (75.0%) in the placebo arm (OR: 0.94; 95% CI: 0.72, 1.23; *P* = 0.671). Common general complaints included gastrointestinal disturbances, skin and eye problems, respiratory and urinary tract infections, and dental caries/abscesses. The prevalence of common complaints did not differ between the groups. Among women with positive pregnancy tests, 6 of 168 (3.6%) taking UNIMMAP and 3 of 181 (1.7%) taking placebo had a positive malaria film during antenatal or perinatal review (OR: 2.20; 95% CI: 0.54, 8.90; *P* = 0.271). Pre-eclampsia was detected in 4 of 203 women (2.0%) in the UNIMMAP arm and 3 of 212 (1.4%) in the placebo arm (OR: 1.40; 95% CI: 0.31, 6.34; *P* = 0.662). At delivery 11 of 177 (6.2%) in the UNIMMAP arm were hypertensive compared with 10 of 186 (5.4%) in the placebo arm (OR: 1.16; 95% CI: 0.48, 2.82; *P* = 0.733).

Neonatal mortality data were available for 325 of 376 (86.4%) live-born singleton infants. There were 5 neonatal deaths in 160 births (3.1%) in the UNIMMAP arm and 4 neonatal deaths in 165 births (2.4%) in the placebo arm (OR: 1.30; 95% CI: 0.34, 4.92; *P* = 0.701). Three nonpregnant women died of unknown causes (2 UNIMMAP, 1 placebo), and one pregnant woman receiving placebo died after antepartum hemorrhage.

## DISCUSSION

To our knowledge, this is the first trial to assess the impact of periconceptional micronutrient supplementation on placental function. Women who took UNIMMAP during early pregnancy had lower uterine vascular resistance indexes than those who took placebo, but this effect was not mediated through a detectable change in placental endothelial function and did not affect transplacental transfer capacity, placental endocrine function, rates of fetal growth, or birth outcomes.

We observed no effect of micronutrient supplementation on the midgestational plasma concentrations of PAI-1 or PAI-2 or their ratio. Because the sample size required to assess a 20% change in this outcome was not achieved, we cannot exclude the possibility of a type II error in our analysis. However, given the CIs of the sample distribution of PAI ratios, other explanations for a null effect are more plausible. In the previous trial, urban British women at high risk of pre-eclampsia who were supplemented with pharmaceutical doses of vitamins C (1000 mg/d) and E (400 IU/d) had a 21% reduction in PAI-1:PAI-2 compared with those taking placebo (27). We supplemented rural Gambian women, in whom the incidence of pre-eclampsia was particularly low (1–2%), with the Recommended Dietary Allowances of 15 micronutrients. The geometric mean PAI ratio in our sample remained small throughout pregnancy as a function of low systemic endothelial activation, making it a poor discriminator of placental function in our population.

Periconceptional UNIMMAP supplementation reduced the indexes of uterine-artery vascular resistance (UtAPI and UtARI; effect size ∼0.25 SD). The effects were small and of no clinical significance, but they support our hypothesis that periconceptional micronutrient status can modulate placental vascular function.

In early pregnancy the uterine-artery Doppler waveform is characterized by a high-resistance profile, transforming to one of low resistance by 24 wk of gestation in normal pregnancies. The persistence of high-resistance waveforms is predictive of subsequent pre-eclampsia, FGR, and placental abruption (30). In a large Dutch cohort, self-reported periconceptional folic acid supplementation was associated with lower utero-placental vascular resistance, although the effect was also small (31). Folic acid could facilitate the epigenetic modulation of early placental growth by acting as a methyl donor during the wave of DNA methylation that takes place shortly after conception (32). In a small subset of women from this study we showed that periconceptional UNIMMAP supplementation led to differential cord blood methylation patterns assessed by using both candidate gene (33) and genome-wide (34) approaches.

Supplementation also reduced fetal middle cerebral PSV (effect size ∼0.33 SD), an inverse marker of fetal hemoglobin concentration (26). By delivery, cord hemoglobin concentrations were similar in both groups, but the effect of prenatal UNIMMAP supplementation on other indexes of neonatal erythropoiesis is unknown.

Periconceptional UNIMMAP supplementation had no effect on transplacental transfer of MMA. We are unaware of other studies that used transfer ratios to assess the impact of nutritional interventions on placental transport function. Experimental work in sheep and rats suggests that general periconceptional undernutrition reduces the expression of transporter proteins in the placenta and the rate of nutrient delivery to the fetus (35). Similarly, we observed that conception in the “wet-hungry” season (a period of nutritional austerity) predicted substantially reduced MMA ratios and birth weights.

Few trials have assessed neonatal anthropometric measurements and pregnancy outcomes with periconceptional micronutrient supplementation (5). We did an exploratory investigation of these outcomes as well as a stratified analysis by season. Within the limits of our sample size, we found no significant differences in any of the birth data between the arms of the trial. There were fewer PTBs in the UNIMMAP group, a finding that is consistent with previous observational studies (36, 37), but the number was small, the CI around the effect estimate was wide, and there was no overall change in the median duration of gestation between the groups. Caution is therefore required in the interpretation of this result, which might be a chance finding. Although seasonality was anticipated to be an important effect modifier in the current trial (18), there was no evidence for a differential effect of supplementation on pregnancies conceived during the wet-hungry season.

Previously, maternal hemoglobin concentration in early pregnancy (although not ferritin concentration) was negatively associated with plasma concentrations of hCG and hCL (38). We found no effect of periconceptional UNIMMAP supplementation on midpregnancy production of these placental hormones.

### Strengths and weaknesses of the study

Our study is a novel and detailed investigation of placental function in a rural African population with low HIV seroprevalence but high rates of anemia; the findings are likely to extrapolate to similar populations elsewhere. Strict eligibility screening ensured that most women conceived after a lengthy period of receiving supplementation. There was enthusiastic community participation in the trial, and close integration of staff with the study population ensured high compliance with supplementation and good clinical oversight. Pregnancy dating was robust for the majority of the women and was based on ultrasound assessment.

Because only 258 of the 3206 women who were pre–randomly assigned (8%) to supplementation are included in the analysis of the primary outcome, protection from bias afforded by the randomization cannot be assumed. Table 1 suggests that of those women who conceived during the trial and were therefore eligible for analysis, more of those in the placebo arm had accessed secular education, although there were no other significant differences identified. There was a loss of data from migration and late booking for antenatal care. Women in traditional societies often delay pregnancy disclosure (39), and 37% of women with a confirmed pregnancy failed to attend the primary endpoint at 18–22 wk of gestation. The resulting loss of power was reflected in the wide CIs around the small increase in PAI-1:PAI-2, estimated with UNIMMAP at 18–22 wk.

Malaria was a potential effect modifier in our study, and we did not examine placental histology to assess this in detail. There was no evidence for a differential effect of supplementation in the wet season, all women had directly observed intermittent preventative treatment for malaria, and rates of peripheral parasitemia were very low throughout the study, suggesting a limited impact from this infection.

### Conclusions

Experimentally, our data suggest that placental vascular function may be modifiable by periconceptional nutritional supplementation in humans, but the therapeutic and public health implications of this finding are unclear. Previously reported effects of prenatal MMS on fetal growth are unlikely to have been mediated through measurable changes in placental vascular, endothelial, transport, or endocrine functions but rather via alternative mechanisms (40).

## Footnotes

6 Abbreviations used: ANC, antenatal clinic; FGR, fetal growth restriction; hCG, human chorionic gonadotropin; hPL, human placental lactogen; LMP, last menstrual period; MCA, middle cerebral artery; MMA, maternal measles antibody; MMS, multiple-micronutrient supplementation; MRC, Medical Research Council; MUAC, midupper arm circumference; NM, nurse-midwife; PAI, plasminogen activation inhibitor; PSV, peak systolic velocity; PTB, preterm birth; RVTH, Royal Victoria Teaching Hospital; TBA, traditional birth attendant; UNIMMAP, UNICEF/WHO/United Nations University international multiple micronutrient preparation; UtA, uterine artery; UtAPI, uterine-artery pulsatility index; UtARI, uterine artery resistance index.
